# The Patient's perspective on radiation therapy for anal cancer: Evaluation of expectations and stigma

**DOI:** 10.1002/cnr2.1908

**Published:** 2023-10-11

**Authors:** Basil H. Chaballout, Eric M. Chang, Narek Shaverdian, Percy P. Lee, Phillip J. Beron, Michael L. Steinberg, Ann C. Raldow

**Affiliations:** ^1^ School of Medicine Greenville University of South Carolina Greenville South Carolina USA; ^2^ School of Medicine Oregon Health and Science University Portland Oregon USA; ^3^ Memorial Sloan Kettering Cancer Center New York New York USA; ^4^ Cancer Treatment Centers of America Cancer Treatment Centers of America Phoenix Arizona USA

**Keywords:** anal cancer, patient expectation, patient perspective, radiation oncology, stigma

## Abstract

**Background:**

Little is known regarding anal cancer patients' perspectives on undergoing radiation therapy. Additionally, the stigma surrounding anal cancer diagnosis warrants a better understanding of the barriers to complete disclosure in patient‐healthcare team interactions.

**Methods:**

Included patients had squamous cell carcinoma of the anus treated with definitive chemoradiation (CRT) from 2009 to 2018. Survey questions were adapted from the European Organization for Research and Treatment of Cancer Quality of Life Questionnaire and Discrimination and Stigma Scale.

**Results:**

A total of 46 anal cancer patients who underwent CRT were surveyed, of which 72% responded. 73% of respondents indicated little to no pre‐treatment knowledge of CRT. 70% reported overall short‐term effects as worse than expected, most commonly with bowel habits (82%), energy (73%), and interest in sexual activity (64%). 39% reported overall long‐term effects to be worse than expected, most commonly with changes to bowel habits (73%), sexual function (67%), and interest in sexual activity (58%). However, 94% agreed they were better off after treatment. Regarding stigma, a subset reported hiding their diagnosis (12%, 24%) and side effects (24%, 30%) from friends/family or work colleagues, respectively, and 15% indicating they stopped having close relationships due to concerns over stigma.

**Conclusions:**

Although patients' perceptions of the severity of short‐term CRT side effects were worse than expectations, the vast majority agreed they were better off after treatment. Targeted counseling on common concerns may improve the anal cancer treatment experience. A notable subset reported stigma associated with treatment, warranting further evaluation to understand the impact on the patient experience.

## 1 INTRODUCTION

Anal cancer cases continues to grow in number with 9090 new cases a year (6070 in women and 3020 in men) and about 1430 deaths (870 in women and 560 in men).^1^ Being that modern advancement in treatment has made this disease largely curable, means of improving quality of life (QOL) post‐treatment is a focal point of research. In effort to maintain patient quality of life and avoid sphincter‐sacrificing surgery, definitive chemoradiation (CRT), consisting of chemotherapy and concurrent radiation therapy (RT), is the standard of care for anal cancer, with multiple randomized trials confirming the efficacy and safety of this approach.^2^, ^3^, ^4^, ^5^ However, patients undergoing CRT may still experience significant treatment‐related acute and late effects potentially impacting both their functional outcome and quality of life.^6^ Multiple studies have evaluated both patient‐ and physician‐reported outcomes of CRT.^7^, ^8^ However, little are known regarding patients' perspectives of their experience during and after treatment. Radiation therapy, in particular, may be associated with significant misconceptions such as intense pain or becoming radioactive, heightening patients' fears of undergoing treatment.^9^, ^10^ Thus, our objective was to further understand and document the patients' perspective of their treatment experience with the goal of improving information for patients and providers.

Anal cancer is associated with HIV and persistent infection of high‐risk human papilloma virus (HPV) strains, with a majority fraction of cases consisting of men with HIV and up to 93% of cancer cases noted to be HPV positive.^11^, ^12^, ^13^ As sexual activity serves as a primary cause of HPV transmission, studies identify stigma as a barrier to uptake of vaccination against the virus.^14^ This association with HPV, compounded by the relatively sensitive nature of the location of the disease, may contribute to stigma surrounding the diagnosis and treatment of anal cancer.^15^ Qualitative studies of anal cancer screening identified stigma and consideration of the anus as hidden/private as barriers to uptake.^16^ How this stigma impacts patients undergoing treatment for anal cancer, however, remains unclear. Stigma and discomfort with the sensitive nature of the disease may inhibit both patients and providers from discussing sensitive issues during consultation, treatment, and follow‐up, causing patients to feel incompletely informed of their treatment and apprehensive when raising adverse effects in clinic.^14^ Therefore, we also sought to understand the ways in which patients' diagnosis and treatment experiences affect stigma. More specifically, we aimed to explore the frequency with which stigma is manifested, primarily by concealment and avoidance of significant relationships. Developing an understanding of patient stigma can greatly enhance the ability of healthcare providers to provide effective support and care for their patients.

## 2 METHODS

### 2.1 Patients

Consecutive patients with squamous cell carcinoma of the anus treated with definitive CRT at the University of California Los Angeles Department of Radiation Oncology from 2009 to 2018 that were without recurrence and with ≥6 months of follow‐up were surveyed.

### 2.2 Instruments and measures

A comprehensive questionnaire was compiled by our team and was adapted from validated patient‐reported questionnaires measuring short‐ and long‐term effects of treatment, including the European Organization for Research and Treatment of Cancer QLQ‐ANL27 and CR29 questionnaires.^17^ The questionnaire responses provided the data of our study, hence, making this a cross‐sectional study. The questionnaire assessed patients' baseline knowledge and fears regarding CRT and asked them to compare their experiences of the short‐term and long‐term side effects of treatment with their initial expectations (see Supporting Information Questionnaire). Specific toxicities were also evaluated, including anal pain, skin changes, limitations to work, limitations to social/recreational activities, fatigue, feeling of illness, anxiety, sadness, disruption to family, changes to pelvic organs, bowel/bladder function, dissatisfaction with body image, and sexual dysfunction. Patients also selected the accuracy level of prewritten statements regarding their pretreatment views about CRT as compared with their sentiment post treatment; an example of such statements being, “Overall, my radiation therapy experience was much harder than I had expected it to be.” Lastly, levels of stigma were evaluated by quantifying how often‐anal cancer survivors attempted to hide/conceal their diagnosis and/or treatment effects from others, including colleagues at work, friends, and family. Questions were adapted the Discrimination and Stigma Scale (DISC‐12).^18^

The survey was administered to 46 patients over a 3 month period. Surveys were mailed or provided to patients in clinic at follow‐up. Data from the surveys were analyzed by means of comparing frequencies of answers selected per question. The UCLA institutional review board has approved this study (IRB #20‐000855), and informed written consent was obtained from all patients.

## 3 RESULTS

### 3.1 Patient clinical characteristics

A total of 46 patients with squamous cell carcinoma of the anus aged <90 years were surveyed at the study institution from November 2018 through January 2019. The response rate was 72% (33 of 46) at a median of 30 months (range, 6.1–109.93 months) after treatment was completed. The median age was 66 years (range 54–88); 61% were female and 12% were HIV positive. The TNM stage distribution was 15% stage I, 33% stage II, and 52% stage III. All patients underwent intensity‐modulated radiation therapy (median total dose 54 Gy, range 48.6–59.4 Gy). The most common concurrently given chemotherapy was 5‐fluorouracil/mitomycin C (94%).Median follow‐up at survey was 30.0 months (range 6.2–116.5).

Majority of responders were female (69%), had completed college (33%) or post graduate (33%) education. The median age was 66 years, and more than half of the responders had stage III disease (51%). Of the 33 responding patients, 4 (12%) patients were HIV positive. All patients underwent IMRT with a median total dose of 54 Gy (48.6–59.4) in 1.8 Gy per fraction. Additionally, all patients received chemotherapy, with 94% receiving 5‐FU/MMC. Table 1 presents the baseline patient characteristics.

**TABLE 1:** Baseline patient characteristics.

Demographic	Number of patients (*N* = 46)	Percentage
Age (years)		
Median	66	
Range	54–88	
Sex		
Male	13	39%
Female	20	61%
Highest education level completed		
High school	8	24%
College	11	33%
Post‐graduate	11	33%
HIV status		
Positive	4	12%
Negative	29	88%
Stage (TNM)		
I	5	15%
II A	10	30%
II B	1	3%
III A	10	30%
III B	0	0%
III C	7	21%
Radiotherapy		
IMRT	33	100%
Boost treatment	33	100%
Chemotherapy		
Any chemotherapy	33	100%
5‐FU/MMC	31	94%

Abbreviation: IMRT, Intensity‐Modulated Radiation Therapy. 5‐FU/MMC, 5‐Fluorouracil/Mitomycin C.

### 3.2 Baseline perceptions and knowledge of RT

**FIGURE 1:**
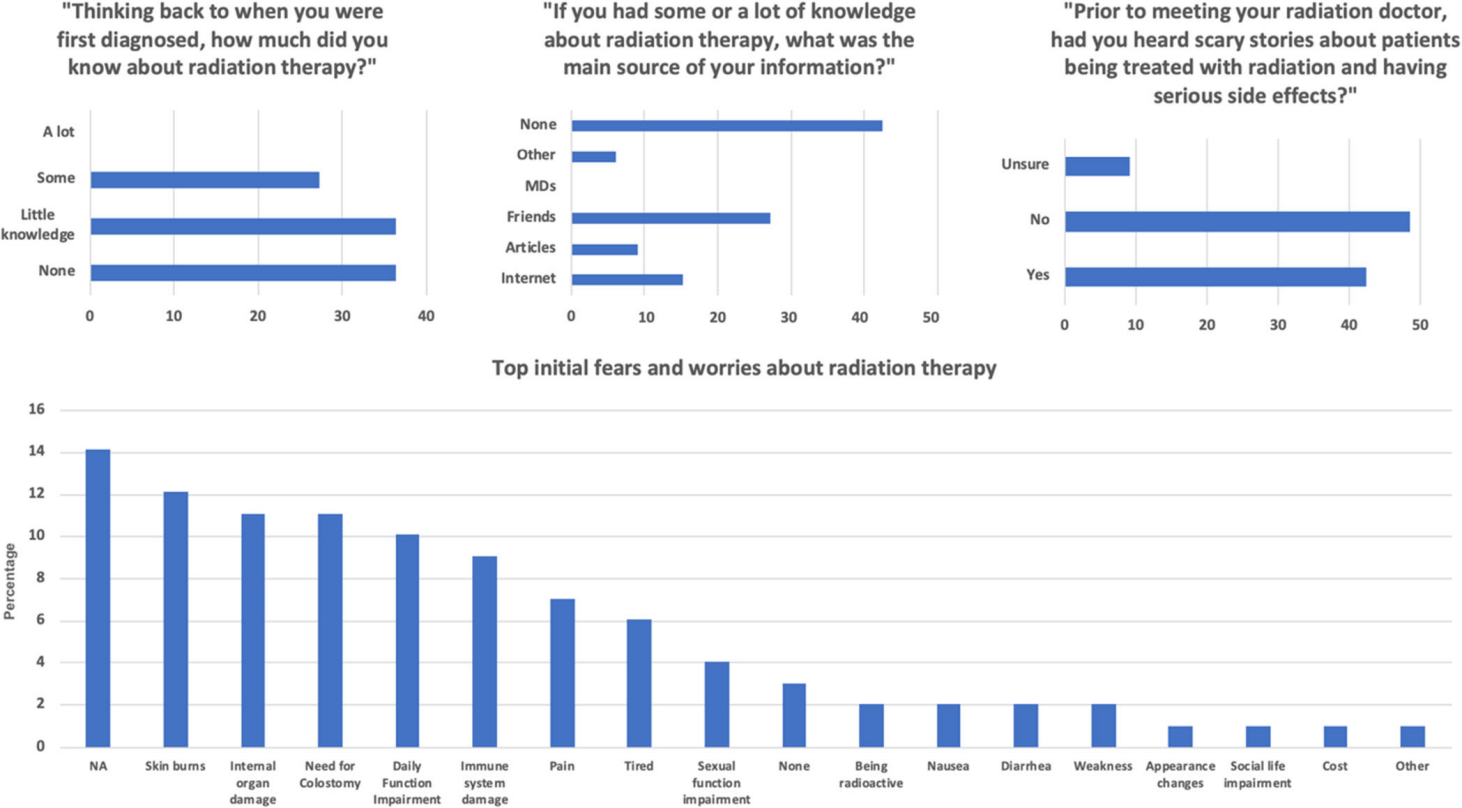
Baseline beliefs and fears regarding radiotherapy (RT) treatment of Anal Squamous Carcinoma prior to actual treatment.

None of the respondents reported having a lot of prior knowledge about RT at diagnosis, while roughly 73% of patients reported they had little to no baseline knowledge. The most common source of information for those patients with some baseline knowledge was friends and family (27%). Approximately, 42% of patients reported previously reading or hearing scary stories about patients having serious side effects from their radiation treatments. When asked regarding baseline fears of RT, most patients did not express any initial fears (Figure 1). Among those who ranked their top fears about RT, the most commonly ranked top initial fear was skin burns (12%), followed by internal organ damage (11%) and need for colostomy (11%). Daily function impairment was ranked highly by 10% of patients, immune system damage by 9% of patients, pain by 7% of patients, fatigue by 6% of patients and sexual function impairment by 4% of patients. The less commonly ranked top fears were being radioactive, nausea, diarrhea, and weakness (ranked by 2% of patients per category), and appearance changes, social life impairment and cost (ranked by 1% of patients).

### 3.3 Beliefs regarding RT post actual treatment experience

**FIGURE 2:**
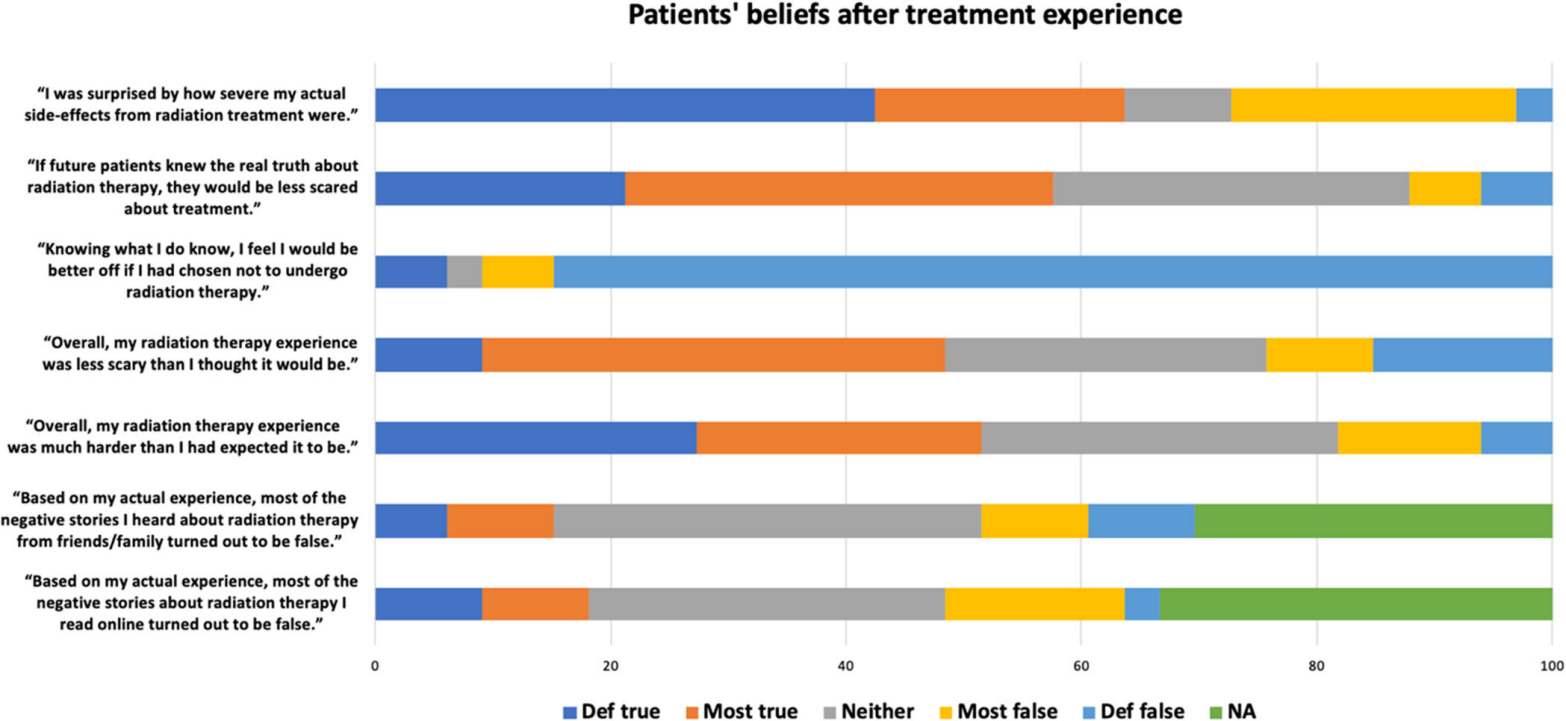
Beliefs after radiotherapy (RT) treatment of Anal Squamous Cell Carcinoma.

Among patients, 18% responded that the negative stories they read about RT prior to treatment were definitely (9%) or mostly (9%) true based on their actual experience Figure 2. Similarly, 15% of patients responded that the negative RT stories from family and friends were in fact true. Approximately 48% of patients reported that their RT experience was less scary than they thought it would be. Of note, 52% of patients found their overall RT experience to be harder than originally expected, with 64% of patients reporting that they were surprised by how severe their actual side‐effects from RT were. However, approximately 91% of patients did not feel they would have been better off without radiation treatment, and more than half (58%) agreed with the statement “if future patient patients knew the real truth about radiation therapy, they would be less scared about treatment”.

### 3.4 Perceptions of acute and long‐term RT toxicity

**FIGURE 3:**
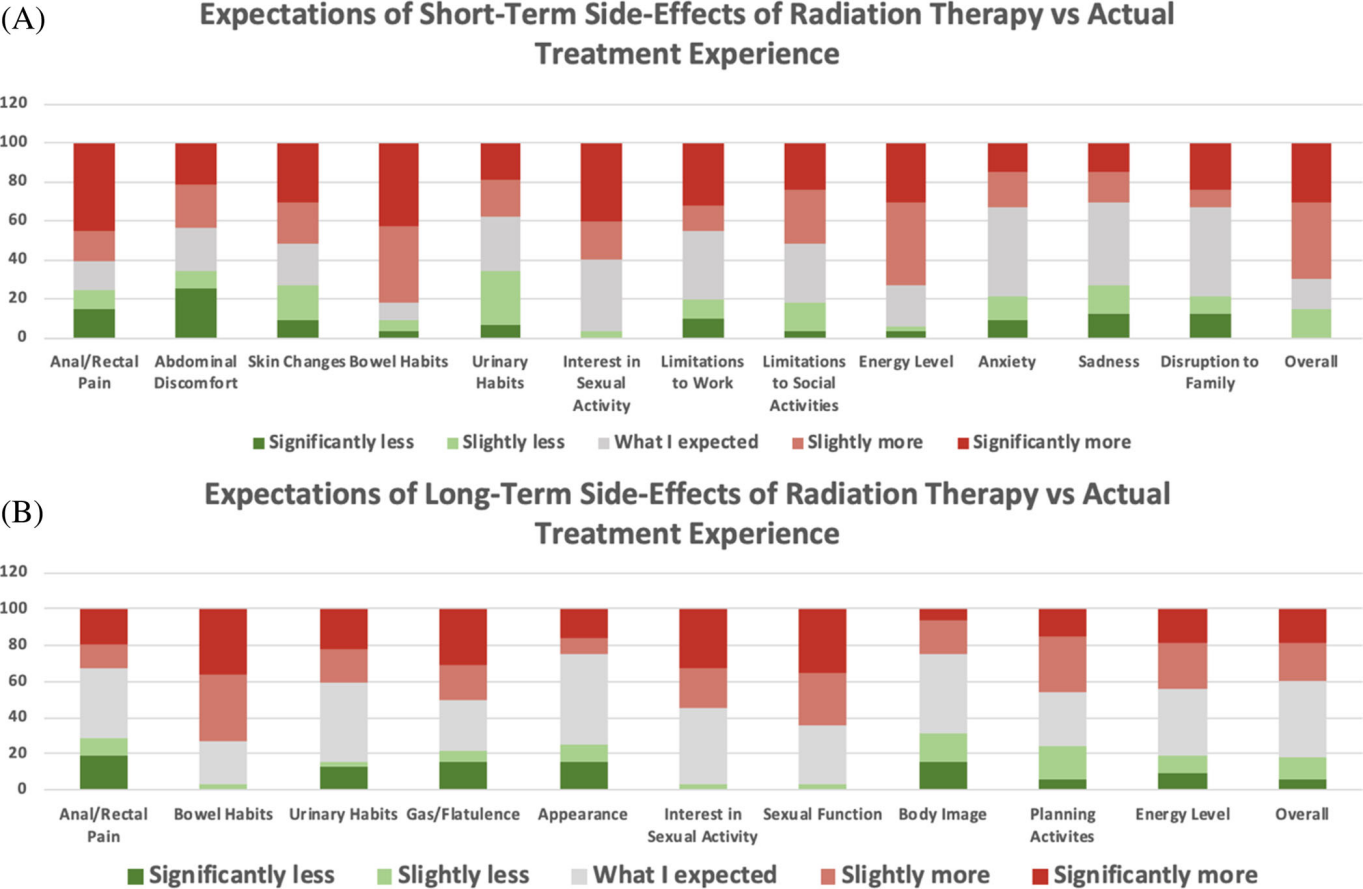
Actual short‐term (A) and long term (B) toxicities compared with initial perceptions.

Among all respondents, approximately 70% responded that their short‐term radiation side effects during RT were either slightly or significantly more severe than expected (Figure 3). Only 15% of patients reported slightly less severe than expected short‐term radiation side effects, with none of the patients reporting significantly less than expected. Approximately 61% of patients found anal or rectal pain severity endured with RT to be slightly or significantly more than expected. Similarly, 52% and 82% of patients reported the short‐term radiation induced skin changes and bowel habits as slightly or significantly more severe than expected. Also, while undergoing RT, 52% and 73% of patients found the limitations to social activities and energy level changes during treatment as slightly or significantly more than expected. On the other hand, 63% and 56% of patients found changes to urinary habits and abdominal discomfort respectively, as less than or as expected during their RT. Limitations to work activities and disruption to friends and family while undergoing RT was experienced as less than or as expected by 54% and 66% of patients respectively. Regarding anxiety, 67% of patients found anxiety during RT as less than or as expected. Similarly, 67% of patients reported less than or as expected amount of sadness during treatment.

In regard to actual long‐term RT side effects, about 61% of patients found overall long‐term RT side effect severity as less than or as expected. Approximately 68% and 59% patients found the severity of anal or rectal pain and changes to urinary habits respectively, to be less than or as expected. Regarding planning activities in advance, 55% of patients found the severity as less than or as expected. Additionally, 75% of patients reported treatment‐area appearance as effected less than or as expected. Similarly, 75% of patients found dissatisfaction with body image from RT to be effected less or as expected. On the other hand, approximately 73% of patients found slightly or significantly more changes to the bowel habits. It should be noted that 55% and 65% of patients reported slightly or significantly more changes to their interest in sexual activity and sexual function respectively. Lastly, the number of reports of more and less or as expected problems with flatulence as a result of RT was the same.

### 3.5 Impact of anal cancer diagnosis

**FIGURE 4:**
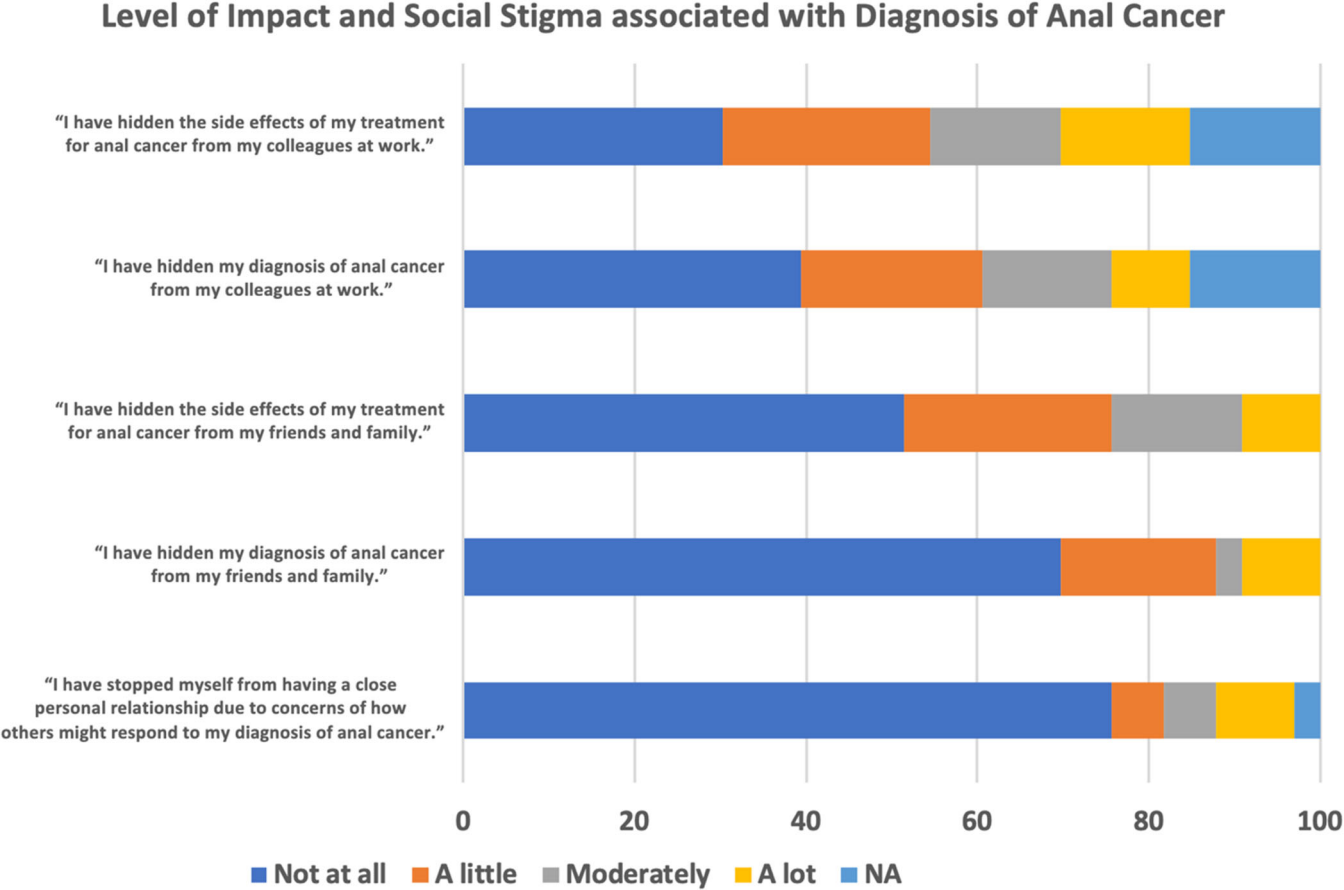
Assessment of level of social stigma associated with diagnosis and treatment of Anal Cancer.

Approximately 76% of all patients disagreed with the statement “I have stopped myself from having a close personal relationship due to concerns of how others might respond to my diagnosis of anal cancer (Figure 4).” 70% of all patients denied hiding their diagnosis of anal cancer, and 52% of patients denied hiding the side effects of their treatment from their family and friends. Approximately, 18% of patients reported hiding their diagnosis from their friends and family a little, and 12% of patients reported doing so moderately or a lot. In addition, 24% of patients reported hiding their side effects from their friends and family a little, 24% of patients reported doing so moderately or a lot. On the other hand, 40% of patients reported that they did not hide their diagnosis of anal cancer, and 30% of patients did not hide the side effects of their treatment from their colleagues at work. However, 21% reported hiding their diagnosis from colleagues at work a little and 24% of patients reported doing so moderately or a lot. Also, approximately 24% reported hiding the side effects of their treatment from colleagues at work a little, while 30% reported doing so moderately or a lot.

## 4 DISCUSSION

The positive prognosis of anal cancer lends a great deal of focus to post‐treatment QOL. Recent studies have explored how additional factors, such as patient perception of illness/treatment, can affect QOL or a patient's overall experience.^9^, ^19^, ^20^ Our study aimed to gather a more comprehensive understanding of how patient's perceive anal cancer treatment and its side effects before and after their treatment experience. We anticipate our findings will help equip physicians with the necessary adjustments in communication and education strategies, and thereby enhance overall patient experience throughout treatment.

The responses acquired in the current study demonstrate that most patients (73%) came into treatment with little to no baseline knowledge of CRT. Nearly all patients (91%) agreed that they were better off having gone through radiation treatment. Despite this, about half (52%) of patients perceived CRT to be harder than originally expected. More than two‐thirds (70%) of patients rated overall severity of acute toxicity to be more than expected. Patients reported system‐specific side effects that were more than expected as well. Regarding the GI system, they were acute and long‐term changes in bowel habits (82% and 73% respectively) and acute anal/rectal pain (61%). Regarding the genitourinary (GU) system, they were long term interest in sexual activity (55%) and sexual function (65%). Regarding overall/other systems, they were acute changes in skin (52%), social activities (52%), and energy levels (73%).

The other half of patients perceived CRT to be less difficult than expected. Regarding long‐term toxicity, more than half (61%) of patients rated overall severity to be less than or as expected. There were system‐specific side effects that patients reported experiencing less than they expected as well. In the GI system, they were acute abdominal discomfort (56%) and long‐term anal/rectal pain (68%). In the GU system, they were acute and long‐term changes in urinary habits (63% and 59% respectively). And in overall/other systems they were acute limitations to work activities (54%), acute limitations to friends/family (66%), acute sadness during treatment, and long term changes in activity planning, appearance of treatment area, and body image dissatisfaction (55%, 75%, and 75% respectively).

More than half of patients agreed that it would help future patients to know the “real truth about radiation therapy,” referring to a desire to have more accurate expectations about treatment rather than preconceived misconceptions prior to treatment or treatment counseling. Pre‐treatment education has been shown to reduce anxiety and increase self‐efficacy in patients receiving radiation.^21^ Increased self‐efficacy has been associated with a higher quality of life.^22^ Since it has been shown that many patients are dissatisfied with pre‐treatment education,^23^ improving this aspect of overall treatment may lead to an overall better patient experience. Additionally, the discrepancy between perception and experience highlights an opportunity for improved pre‐treatment counseling to establish realistic and consistent patient expectations. This is especially important, as it has been noted that the expectation of a side effect may have more bearing on a patient's experience than the absolute severity itself.^19^ Focusing on this can act to also strengthen the patient‐physician alliance. In fact, it was found that the manner in which patient's perceived their inter‐provider communication and overall experience is an important predictor of patient outcomes.^20^

Our study also aimed to explore how stigma regarding anal cancer may influence patient perception. The noted discrepancy of anal cancer knowledge may in fact be influenced by the stigma regarding anal cancer. Stigma has been documented as a barrier to anal cancer screening in general.^14^ Data for the study demonstrated that several patients indeed were hesitant to discuss details regarding their anal cancer with friends and family. Of note, 60% of patients did not deny hiding their diagnosis from colleagues at work, and 30% of patients did not deny hiding their diagnosis from friends and family. Additionally, 70% of patients did not deny hiding side effects of treatment from colleagues at work, and 48% of patients did not deny hiding side effects of treatment from family and friends. While most patients did not withhold information regarding their condition, it is important to recognize that the remainder of patients did indicate that such information was kept private. This demonstrates that there may be a lingering reluctance or discomfort regarding discussing anal cancer that is contributing to the gaps in knowledge discussed above. It has previously been suggested that clinicians increase their education about anal cancer to serve as “point‐persons” for educating patient populations.^14^ Identifying patients at risk may serve as the first step for clinicians who take on this role.

There are several limitations to this study. Despite having a diverse patient population, all of the patients were treated at the same institution. This may have had geographic and socioeconomic influences that biased the results. Furthermore, specific ethnicity data was not included in our baseline characteristics or in our assessment. Additionally, this study was a cross‐sectional study, which overall allowed for a momentary assessment of these outcomes. Due to the nature of using patient‐reported surveys to collect data, recall bias also may have played a role in the responses. However, there was comparative agreement across this sample of patients suggesting this bias may have had only minimal impact. Another limitation is that patients were not interrogated on their specific experience with chemotherapy, which does not allow us to parse out how much of their CRT experience was influenced by the chemotherapy portion of their treatment as opposed to just the radiation. Therefore, it is important to remember that this study reflects experience of radiation therapy with no distinction on which portion of CRT influenced patients the most. Lastly, the response rate of 72% limited the data to 33 patients. Further studies are recommended to further corroborate these results. A nationwide, collaborative survey assessing multiple cancer treatment perceptions versus experiences to solidify and expand these findings may be of benefit.

Nonetheless, based on all the aforementioned data, it remains that targeted counseling on common concerns may improve the anal cancer treatment experience. A notable subset reported stigma associated with treatment, warranting further evaluation to understand the impact on the patient experience. Although patients' perceptions of the severity of short‐term CRT side effects were inferior to expectations, however, the vast majority agreed they were better off after treatment.

## AUTHOR CONTRIBUTIONS

**Basil H. Chaballout:** Conceptualization (equal); data curation (equal); formal analysis (equal); funding acquisition (equal); investigation (equal); methodology (equal); project administration (equal); resources (equal); software (equal); supervision (equal); validation (equal); visualization (equal); writing – original draft (lead); writing – review and editing (lead). **Eric Chang:** Conceptualization (equal); data curation (equal); formal analysis (equal); funding acquisition (equal); investigation (equal); methodology (equal); project administration (equal); resources (equal); software (equal); supervision (equal); validation (equal); visualization (equal); writing – original draft (lead); writing – review and editing (lead). **Narek Shaverdian:** Conceptualization (equal); data curation (equal); formal analysis (equal); funding acquisition (equal); investigation (equal); methodology (equal); project administration (equal); resources (equal); software (equal); supervision (equal); validation (equal); visualization (equal); writing – original draft (equal); writing – review and editing (equal). **Percy P. Lee:** Conceptualization (equal); data curation (equal); formal analysis (equal); funding acquisition (equal); investigation (equal); methodology (equal); project administration (equal); resources (equal); software (equal); supervision (equal); validation (equal); visualization (equal); writing – original draft (equal); writing – review and editing (equal). **Phillip J Beron:** Conceptualization (equal); data curation (equal); formal analysis (equal); funding acquisition (equal); investigation (equal); methodology (equal); project administration (equal); resources (equal); software (equal); supervision (equal); validation (equal); visualization (equal); writing – original draft (equal); writing – review and editing (equal). **Michael Steinberg:** Conceptualization (equal); data curation (equal); formal analysis (equal); funding acquisition (equal); investigation (equal); methodology (equal); project administration (equal); resources (equal); software (equal); supervision (equal); validation (equal); visualization (equal); writing – original draft (equal); writing – review and editing (equal). **Ann Raldow:** Conceptualization (equal); data curation (equal); formal analysis (equal); funding acquisition (equal); investigation (equal); methodology (equal); project administration (equal); resources (equal); software (equal); supervision (equal); validation (equal); visualization (equal); writing – original draft (equal); writing – review and editing (equal).

## CONFLICT OF INTEREST STATEMENT

The authors declare no conflicts of interest.

## ETHIC STATEMENT

The UCLA institutional review board has approved this study (IRB #20‐000855), and informed written consent was obtained from all patients.

## PATIENT CONSENT

The study was explained to the patients, and their subsequent participation in the surveys served as their informed consent.

## Supporting information


Data S1: supporting Information.

## Data Availability

The data that support the findings of this study are available from the corresponding author upon reasonable request.
